# 12q14 microduplication: a new clinical entity reciprocal to the microdeletion syndrome?

**DOI:** 10.1186/s12920-019-0653-x

**Published:** 2020-01-03

**Authors:** Sofia Dória, Daniela Alves, Maria João Pinho, Joel Pinto, Miguel Leão

**Affiliations:** 1 Genetics Service, Department of Pathology, Faculty of Medicine, University of Porto, Alameda Professor Hernâni Monteiro, 4200 Porto, Portugal; 2 Instituto de Investigação e Inovação em Saúde – i3S, Universidade do Porto, Porto, Portugal; 3 Department of Pediatrics, São João Hospital Centre – CHSJ, Porto, Portugal; 4 Department of Medical Genetics, São João Hospital Centre, - CHSJ, Porto, Portugal

**Keywords:** 12q14 microduplication, 12q14 microdeletion, Overgrowth, Obesity, *HMGA2*

## Abstract

**Background:**

12q14 microdeletion syndrome is characterized by low birth weight and failure to thrive, proportionate short stature and developmental delay. The opposite syndrome (microduplication) has not yet been characterized. Our main objective is the recognition of a new clinical entity - 12q14 microduplication syndrome. - as well as confirming the role of HMGA2 gene in growth regulation.

**Case presentation:**

Array Comparative Genomic Hybridization (CGH), Karyotype, Fluorescence in situ Hybridization, Quantitative-PCR analysis and Whole exome sequencing (WES) were performed in a girl presenting overgrowth and obesity. Array CGH identified a 1.5 Mb 12q14.3 microduplication involving HMGA2, GRIP1, IRAK3, MSRB3 and TMBIM4 genes. Karyotype and FISH showed that duplication was a de novo insertion of 12q14.3 region on chromosome 9p resulting in an interstitial microduplication. Q-PCR confirmed the duplication only in the proband. WES revealed no pathogenic variants.

**Conclusions:**

Phenotypic comparison with patients with 12q14 microdeletion syndrome showed a reciprocal presentation, suggesting a phenotypically recognizable 12q14 microduplication syndrome as well as confirming the role of HMGA2 gene in growth regulation. It is also indicative that other genes, such as IRAK3 and MSRB3 might have of role in weight gain and obesity.

## Background

Copy number variations on chromosome 12q14 have been mainly reported as a microdeletion syndrome (OMIM #166700) first described by Menten et al. in 2007 and classified as an autosomal dominant bone dysplasia. The patients showed failure to thrive in infancy evolving to proportionate short stature, mild intellectual disability and osteopoikilosis [1]. Other authors reported similar cases with 12q14 deletions, including total or partial deletions of *HMGA2*, *GRIP1* and in some cases *LEMD3, IRAK3* and *TMBIM4* genes [2–6]. Duplications on 12q14.3 have not yet been reported and the previously reports involving *HMGA2* gene gains of function were acquired rearrangements related with potentially malignant conditions. The majority were *HMGA2* fusions with partner genes and subsequently formation of chimeric transcripts with gain of *HMGA2* gene function and tumor formation [7–11].

Previous reports on microdeletions within 12q14 region ranged from 1.83 Mb to 10.12 Mb with a 378Kb smallest region of overlap containing the *HMGA2* and *IRAK3* genes [3–12]. Several authors confirmed the association between growth impairment and loss of *HMGA2* gene as a common feature in the 12q14 microdeletion syndrome [3–5]. There are no publications about 12q14 microduplication opposite syndrome. Here we describe for the first time a patient with a 1.5 Mb 12q14 insertion on chromosome 9p resulting in a microduplication identified by array comparative genomic hybridization (array-CGH). The patient presented at our office at 8 months of age with mild dysmorphic features, overgrowth and obesity. The duplicated region overlaps the previously reported deletions including *HMGA2, GRIP1, IRAK3, MSRB3* and *TMBIM4* genes and the phenotype associated with the duplication apparently mirrors the microdeletion syndrome phenotype.

## Case presentation

**Fig. 1:**
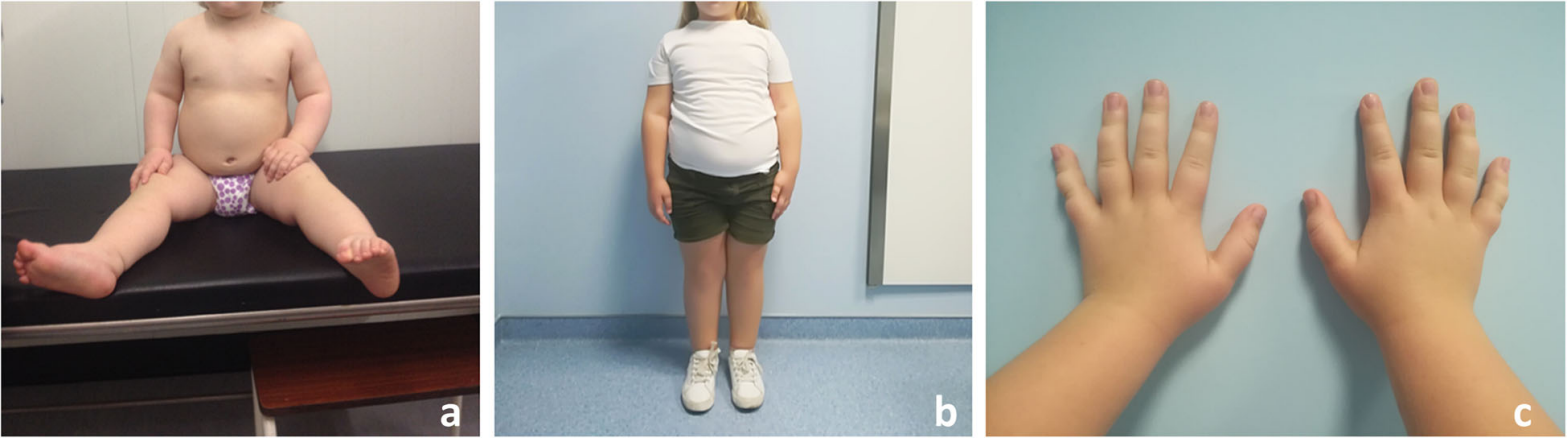
**a** Patient at 18 months of age showing obesity; **b** Patient at present with. 5 years and 5 months weighting 52Kg; **c** Edematous and tapering appearance of the fingers

Our patient was born to non-consanguineous 37 year-old Human Immunodeficiency Virus (HIV) positive mother and 22 year-old healthy father. She has an 18 year-old healthy sister on her mother side. Anti-retroviral medication was carried out throughout both pregnancy and delivery according to national guidelines, and this treatment was not complicated by any adverse effects. The girl was born by caesarean section at 38 and 2/7 weeks, and Apgar scores were 9 and 10 at 1st and 5th minutes respectively. Birth weight was 4015 g (P95), length 50.5 cm (P75–90) and OFC 36 cm (P95). She was admitted to the neonatal intensive care unit soon after birth for moderate respiratory distress. She had mild hypotonia and feeding difficulties. Head ultrasound was normal. Supraventricular extrasystoles conditioning tachyarrhythmia were noted on electrocardiogram, and she received propranolol treatment transiently. Echocardiogram was normal. No other anomalies were noted. Upon follow up after discharge she was noted to have increased growth parameters with marked obesity, weighing 12.7Kg at six months (+ 6.7SD). At 8 months she was referred for genetic evaluation. She had developed mild dysmorphic features, with slight bilateral ptosis, scarce eyelashes, anteverted nostrils and macroglossia. She had inverted nipples and increased inter nipple distance. Both hands and feet seemed edematous and she had tapering fingers. She had normal skin. Follow up studies at 12 and 18 months showed cholesterol, apolipoprotein A1 and B, and endocrine studies (cortisol, C peptid and insulin levels, growth hormone and thyroid function tests) were normal. Serological markers for HIV were negative. She had no signs of urogenital abnormalities, visceromegaly or lipomatosis on abdominal ultrasounds. Radiologic studies did not show any signs of skeletal dysplasia. At 18 months her weight was 20.2Kg (+ 5.1SD), height was 91 cm (+ 3.6SD), occipitofrontal circumference (OFC) 50 cm (+ 2.8SD) and body mass index (BMI) 24.4 (+ 6.7SD) (Fig. 1a). Bone age - determined by radiography of the hand and wrist - was advanced, equivalent to a 28 month-old girl. She had normal hearing and developmental milestones were achieved within the normal timing range. At present with 5 years and 5 months, she weighs 52Kg (+ 5.5SD), her height is 131 cm (+ 3.9SD), her OFC 55 cm (+ 3.6SD estimated from Zscore at 60 months of age) and BMI 30.3 (+ 5.8SD estimated from Zscore at 60 months of age) (Fig. 1b). Her hands measure 15.5 cm, the palm 9 cm and the middle finger 6.5 cm. The edematous and tapering appearance of the fingers persists (Fig. 1c). She has normal intelligence and a very good memory.

An array-CGH was carried out using Agilent SurePrint G3 Human Genome microarray 4 × 180 K platform (Agilent Technologies, Santa Clara, CA) according to manufacturer recommendations Results were analyzed using Cytogenomic software 2.9.2.4 and Human Genome 19 (GRCh37) assembly.

Copy number quantification was carried out by quantitative real time PCR (qPCR) on a StepOnePlus™ Real-Time PCR System (Life Technologies Corporation, California, USA) using the taqman Hs01969532_cn probe (Applied Biosystems) locate within exon 6 of *HMGA2* gene. Applied Biosystems CopyCaller v2.0 software was used to calculate the copy number probe and the confidence of the statistical method.

Conventional G Banding with Trypsin and Leishman (GTL banding) and Fluorescence in situ hybridization (FISH) studies were performed on the proband and progenitors peripheral blood lymphocytes using standard protocols and according to the International System for Human Cytogenetic Nomenclature (ISCN) 2016 [13].

Centromeric FISH probes for chromosomes 9 (*green* color) and 12 (*aqua* color) (Abbot®) and also a SureFISH probe specific for 12q14.3 *HMGA2* gene region (*red* color) (cat. # G100381R8, Agilent Technologies, Santa Clara) were used.

Whole exome sequencing (WES) was performed as a trio on proband and progenitors using Agilent SureSelect Human All Exon V6 and Illumina Hiseq2000 plataform. GRCh37 was used as Human Genome Reference.

Standard deviation scores were calculated according to the World Health Organization (WHO) Growth Reference Data Z-scores (Girls: Weight-for-age birth to 2 years/5 to 10 years; Length-for-age birth to 2 years/Height-for-age 5 to 19 years; Head circumference-for-age birth to 5 years; Body mass index-for-age birth to 5 years).

**Fig. 2:**
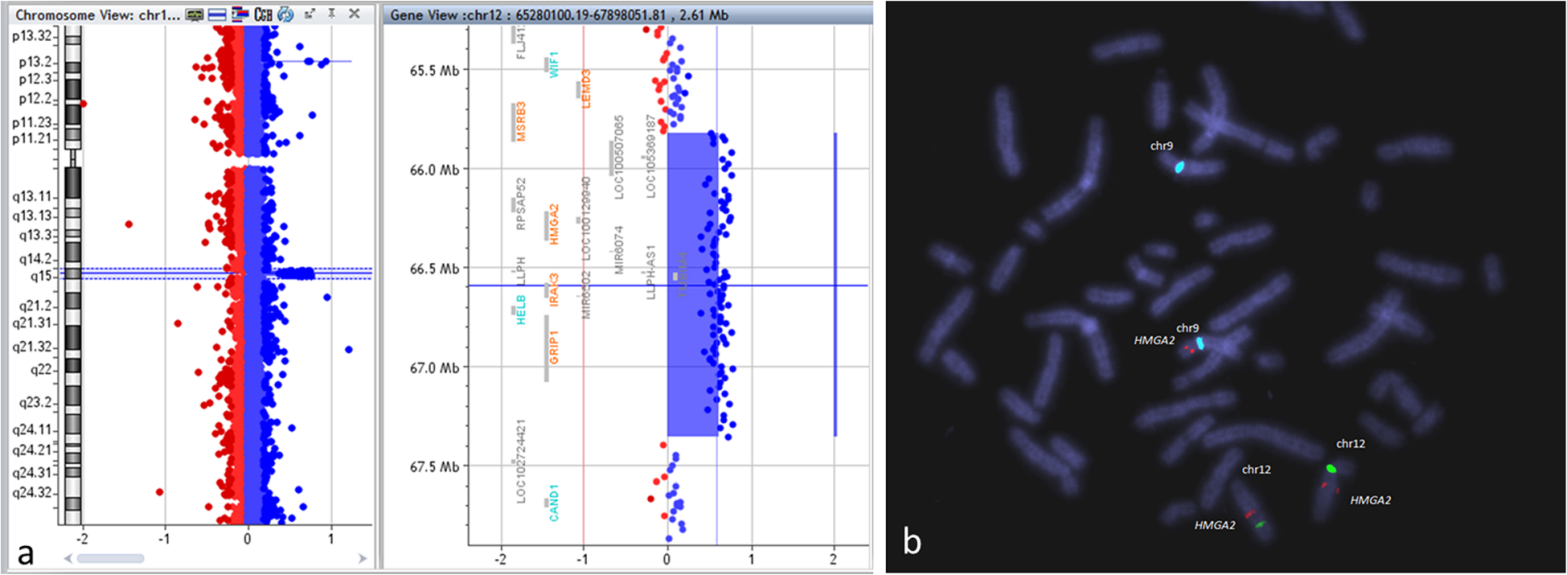
**a** Array CGH profile of the chromosome 12, showing the amplified region 12q14.3 (chr12:65,822,792-67,355,360; NCBI hg19). **b** FISH results showing insertion of *HMGA2* gene into chromosome 9p; SureFISH probe specific for 12q14.3 *HMGA2* gene region in red and centromeric probes for chromosomes 9 and 12 in aqua and green, respectively

Array CGH analysis revealed a 12q14.3 duplication spanning about 1,5 Mb with genomic positions at 65822792_67355360 (hg19; GRCh Build 37.1, February 2009). The duplication region contains 8 genes, including *HMGA2, IRAK3, GRIP1, MSRB3, TMBIM4, HELB, RPSAP52 and LLPH* (Fig. 2a). In order to validate the array CGH result and further evaluate the parents, qPCR was performed. Results confirmed the *HMGA2* gene copy number gain in the proband and showed a normal copy number for the parents. This result is compatible with a de novo 12q14.3 duplication.

Proband and progenitors karyotypes were normal. FISH studies carried out in the patient with a specific probe for *HMGA2* gene revealed one signal in each q14.3 arm of both chromosomes 12 and a third signal on chromosome 9p arm (Fig. 2b). FISH studies in the progenitors’ lymphocytes using the same probes were normal (data not shown). This is compatible with a de novo insertion of 1.5 Mb 12q14.3 region on chromosome 9p resulting in a microduplication 12q14.3, described as follows: 46,XX.ish ins(9;12)(p2?2;q14.3)(HMGA2+).arr[GRCh37] 12q14.3(65822792_67355360)× 3.

Additional studies using WES for patient and progenitors reveled no pathogenic variants.

## Discussion and conclusions

Unlike the 12q14 microdeletion syndrome, the effect of a microduplication in the same region in humans has not yet been published.

In the first description of 12q14 microdeletion syndrome in 2007 Menten et al. reported three patients with similar main phenotypic characteristics, the most distinctive of which was osteopoikilosis [1]. Other features were failure to thrive, proportionate short stature and in some cases intellectual disability. This 12q14 microdeletion encompassed a 3.44 Mb region and included the *HMGA2, GRIP1, LEMD3, MSRB3* and *TMBIM4* genes [1]. Using array CGH, Buysse et al. reported in 2009 two additional patients with a shortest overlapping region of 2.61 Mb including *HMGA2*, *GRIP1* and *LEMD3* genes. Subsequently, other authors published additional patients carriers of the microdeletion syndrome, the majority presenting short stature, failure to thrive and developmental delay [2, 6, 12].

The *HMGA2* gene (High mobility group protein 1-C) encodes a protein that belongs to the non-histone chromosomal high mobility group (HMG) protein family. These proteins function as architectural factors and contain structural DNA-binding domains that may act as transcriptional regulating factors. The *HMGA2* gene consists of five exons spanning a genomic region of 160 kb. Exons 1–3 encode A-T hook domains that bind to the minor grove of AT- rich DNA. In humans, haploinsufficiency of the*HMGA2* gene is capable of causing a poor growth phenotype with failure to thrive and short stature, as seen in the 12q14 microdeletion. The association between growth retardation and short stature and the loss of the *HMGA2* gene has been established [1–3, 5, 12]. Buysse et al. described one patient carrier an isolated deletion of *HMGA2 *presenting exclusively short stature and none of the other 12q14 microdeletion syndrome features. *HMGA2* deletion was also present in this patient’s mother and maternal grandmother, and also in a maternal aunt, all presenting short stature as well. The patient and his relatives therefore did not fulfill the criteria described for a 12q14 microdeletion syndrome, suggesting instead a strong association between the *HMGA2* gene deletion and short stature [3].

Ligon et al. by other side described a patient with overgrowth and advanced bone age who had a de novo pericentric inversion of chromosome 12, with breakpoints at p11.22 and q14.3, apparently truncating *HMGA2* [10]. The patient showed in addition a significant different set of phenotypic characteristics, including megaepiphyseal dysplasia, dental anomalies, cerebellar tumor and multiple subcutaneous lipomas, which could suggest alternative diagnosis such as PTEN-Hamartoma or Proteus syndrome.

In vivo data shows that mice with homozygous *hmga2* inactivation are born with reduced body size and extremely decreased fat levels [14] whereas mice expressing a truncated hmga2 develop gigantism and lipomatosis [15]. It is generally assumed that the altered expression of *HMGA2* is an early event in the pathway to tumor formation. Normally, the expression of *HMGA2* is restricted to undifferentiated mesenchymal cells. Chromosomal rearrangements frequently result in the activation of the normally silent *HMGA2* allele in terminally differentiated cells. It has been suggested that such activation can lead to mesenchymal tumor formation [7, 16]. It has also been postulated that the gain of function occurs because of the addition of regulatory domains that could contribute to the tumor formation [7]. Additionally it has been shown that the *HMGA2* 3′-UTR contains target sites for the *let*-7 miRNA and some rearrangements may lead to increased levels of *HMGA2* protein due to the loss of mi-RNA-mediated repression [17].

Ashar et al. suggest that in lipomas *HMGA2* is expressed either solely from the translocated allele associated with a gain of function or from the nondisrupted allele when the disrupted allele does not acquire a regulatory domain [7, 16]. Although *HMGA2* has been identified as the target gene of in mesenchymal tumors, namely in lipomas, the exact mechanism by which *HMGA2* overexpresssion drives benign tumorigenesis in a target cell is still widely unknown [18].

According to Heriksen et al. *HMGA2* seems to act in a narrow window early in the adipogenesis where it is involved in expanding the population of preadipocytes and keepping the cells in an undifferentiated state. The authors concluded that in an in vitro model both wild type and truncated *HMGA2 *resulting in an overexpression of the gene abrogated growth inhibition and adipogenesis [19], which seems inconsistent with the fact that transgenic mice overexpressing truncated *HMGA2* gene develop undifferentiated lipomas and abundant adipose tissue [15]. This could be explained by differences in in vitro or in vivo model systems. In our patient, the presence of an extra complete copy of the *HMGA2* gene rather a rearrangement (truncated *HMGA2* gene) could also involve different regulatory mechanisms. Publications of new constitutional cases with similar phenotypes will be necessary to confirm this possible new entity.

Taking into account the clinical data, all previous reports and the phenotype associated with the microdeletion, the duplication of *HGMA2* with a dosing effect could explain the overgrowth of our patient.

The absence of lipomas or other tumors thus far in our patient remains to be elucidated. Nonetheless, given the patient’s young age these features can still arise later in life, and follow up medical screening is mandatory.

In our patient array CGH revealed the presence of three copies of the *HMGA2* gene and FISH demonstrated that the third copy was inserted on chromosome 9p. The presence of an insertion on p arm of this chromosome could also interfere with gene overexpression or subexpression. Literature revision on genes located chromosome 9p and association for overgrowth or obesity showed no results except a paper suggesting a susceptibility *loci* on 9p22 for adiposity phenotypes [20]. Although unlikely, a position gene effect caused by the insertion itself on chromosome 9, and not by overexpression of the duplicated genes, could not be excluded. It also should be stressed that WES did not show any pathogenic or probably pathogenic variants either in chromosome 9 or 12. Additionally, whole genome sequencing (WGS) instead of WES could help to understand the genomic localization of the 9p breakpoints and to determine if a gene with unknown function is disrupted.

*GRIP1* (OMIM#604597)gene has been implicated in the regulation of glutaminergic and perhaps also GABAergic signaling, both of which are involved in many neuronal mechanisms, and it is known that bialelic mutations of this gene cause Fraser syndrome [21]. Additionally, in vivo studies have found that *GRIP1* is also necessary for dendritic development [22]. Furthermore, heterozygous *GRIP1* variants have been described in patients with neuronal and behavioral autistic phenotypes [23]. Of the 12 patients described with 12q14 microdeletion syndrome involving the *GRIP1* gene only one did not present developmental delay [6]. *GRIP1* is involved in our patient duplication and apart from neonatal hypotonia, development and language have occurred within the normal timing frame.

IRAK3 (OMIM#604459) encodes a member of the interleukin-1 receptor-associated kinase protein family. Members of this family are essential components of the Toll/IL-R immune signal transduction pathways and mutations in this gene have been associated with a susceptibility to asthma [24]. It also has been shown to have decreased expression in obese patients and correlate positively with blood adiponectin. Patients with low levels of adiponectin have lower expression of *IRAK3* and increased obesity [25]. Hulsmans et al. also found that not only *IRAK3* is downregulated in obesity, but also that its expression is increased following weight loss [25]. Clinical consequences of *IRAK3* duplication are not known. Dosing of blood adiponectin was not performed in our patient. As our patient presents severe obesity, implication of *IRAK3* gene cannot be excluded.

*MSRB3* (OMIM#613719) is predominantly located to the endoplastic-reticulum and is thought to play a role in cell growth. Bialelic mutations of this gene cause autosomal recessive deafness 74 [26]. Overexpression of *MSRB3* protects mammalian and Drosophila cells against endoplastic-reticulum stress and has been shown to increase the longevity of Drosophila [27]. These authors have also found evidence suggesting *MSRB3* deficiency induces cell growth inhibition in mouse embryonic fibroblasts. Haploinsufficiency of *MSRB3* could therefore contribute to the growth restriction seen in all patients with 12q14 microdeletion. Correspondingly, duplication of *MSRB3* with a simultaneous gain of function could cause overgrowth, either by inducing growth itself or by not regulating it.

*TMBIM4* is a candidate gene for susceptibility to type 2 diabetes and obesity [28]. As metabolic diseases can arise from changes in gene expression, *TMBIM4* duplication can put our patient at a higher risk of type 2 diabetes.

*LLPH* is an intrinsically disordered protein predicted to function in nucleolus, possibly as a molecular hub for protein-protein interaction and regulating neuronal morphogenesis and synaptic transmission [29]. *HELB* is a DNA Helicase (also known as *HDBB*) and it has been hypothesized that *HELB* is recruited to sites of DNA damage and has a role in recovery from replication stress [30]. Nevertheless, studies in humans regarding the *LLPH* and *HELB* genes have not been published.

Patients with 12q14 microdeletion syndrome present low birth weight and failure to thrive, proportionate short stature and developmental delay, and in some cases osteopoikilosis. Our patient, presenting overgrowth, obesity and tall stature with advanced bone age, is the first described case with 12q14 duplication. Phenotypical comparison of both entities shows that they mirror each other. We propose that this phenotype could represent a new microduplication 12q14 syndrome. WES studies showing no other known pathogenic variants support the causative effect of the duplication. In conclusion, our results strongly support the role of *HMGA2* gene in growth regulation and suggest that other genes, such as *IRAK3 and MSRB3* might have a role in weight gain and obesity.

## Data Availability

All data generated or analyzed during this study are included in this published article. Arrays files are available in https://www.ebi.ac.uk/arrayexpress/experiments/E-MTAB-1234

## Abbreviations

array CGH: Array comparative genomic hybridization
BMI: Body mass index
FISH: Fluorescence in situ hybridization
GTL banding: G Banding with Trypsin and Leishman
HIV: Human Immunodeficiency Virus
ISCN: International System for Human Cytogenetic Nomenclature
OFC: Occipitofrontal circumference
qPCR: Quantitative real time polymerase chain reaction
WES: Whole exome sequencing
WGS: Whole genome sequencing
WHO: World Health Organization

## References

[1] Menten B, Buysse K, Zahir F, Hellemans J, Hamilton SJ, Costa T (2007). Osteopoikilosis, short stature and mental retardation as key features of a new microdeletion syndrome on 12q14. J Med Genet.

[2] Bibb AL, Rosenfeld JA, Weaver DD (2012). Report of a mother and daughter with the 12q14 microdeletion syndrome. Am J Med Genet A.

[3] Buysse K, Reardon W, Mehta L, Costa T, Fagerstrom C, Kingsbury DJ (2009). The 12q14 microdeletion syndrome: additional patients and further evidence that HMGA2 is an important genetic determinant for human height. Eur J Med Genet.

[4] Lynch SA, Foulds N, Thuresson AC, Collins AL, Anneren G, Hedberg BO (2011). The 12q14 microdeletion syndrome: six new cases confirming the role of HMGA2 in growth. European journal of human genetics : EJHG.

[5] Spengler S, Schonherr N, Binder G, Wollmann HA, Fricke-Otto S, Muhlenberg R (2010). Submicroscopic chromosomal imbalances in idiopathic silver-Russell syndrome (SRS): the SRS phenotype overlaps with the 12q14 microdeletion syndrome. J Med Genet.

[6] Takenouchi T, Enomoto K, Nishida T, Torii C, Okazaki T, Takahashi T (2012). 12q14 microdeletion syndrome and short stature with or without relative macrocephaly. Am J Med Genet A.

[7] Ashar HR, Tkachenko A, Shah P, Chada K (2003). HMGA2 is expressed in an allele-specific manner in human lipomas. Cancer Genet Cytogenet.

[8] Bianchini L, Birtwisle L, Saada E, Bazin A, Long E, Roussel JF (2013). Identification of PPAP2B as a novel recurrent translocation partner gene of HMGA2 in lipomas. Genes Chromosom Cancer.

[9] Italiano A, Cardot N, Dupre F, Monticelli I, Keslair F, Piche M (2007). Gains and complex rearrangements of the 12q13-15 chromosomal region in ordinary lipomas: the "missing link" between lipomas and liposarcomas?. Int J Cancer.

[10] Ligon AH, Moore SD, Parisi MA, Mealiffe ME, Harris DJ, Ferguson HL (2005). Constitutional rearrangement of the architectural factor HMGA2: a novel human phenotype including overgrowth and lipomas. Am J Hum Genet.

[11] Schoenmakers EF, Wanschura S, Mols R, Bullerdiek J, Van den Berghe H, Van de Ven WJ (1995). Recurrent rearrangements in the high mobility group protein gene, HMGI-C, in benign mesenchymal tumours. Nat Genet.

[12] Alyaqoub F, Pyatt RE, Bailes A, Brock A, Deeg C, McKinney A (2012). 12q14 microdeletion associated with HMGA2 gene disruption and growth restriction. Am J Med Genet A.

[13] sA MG-JJ, Schmid A (2016). ISCN - An International System for Human Cytogenomic Nomenclature: Karger.

[14] Zhou X, Benson KF, Ashar HR, Chada K (1995). Mutation responsible for the mouse pygmy phenotype in the developmentally regulated factor HMGI-C. Nature..

[15] Battista S, Fidanza V, Fedele M, Klein-Szanto AJ, Outwater E, Brunner H (1999). The expression of a truncated HMGI-C gene induces gigantism associated with lipomatosis. Cancer Res.

[16] Tkachenko A, Ashar HR, Meloni AM, Sandberg AA, Chada KK (1997). Misexpression of disrupted HMGI architectural factors activates alternative pathways of tumorigenesis. Cancer Res.

[17] Mayr C, Hemann MT, Bartel DP (2007). Disrupting the pairing between let-7 and Hmga2 enhances oncogenic transformation. Science.

[18] Thies HW, Nolte I, Wenk H, Mertens F, Bullerdiek J, Markowski DN (2014). Permanent activation of HMGA2 in lipomas mimics its temporal physiological activation linked to the gain of adipose tissue. Obesity (Silver Spring, Md).

[19] Henriksen J, Stabell M, Meza-Zepeda LA, Lauvrak SA, Kassem M, Myklebost O (2010). Identification of target genes for wild type and truncated HMGA2 in mesenchymal stem-like cells. BMC Cancer.

[20] Aberg K, Dai F, Sun G, Keighley ED, Indugula SR, Roberts ST (2009). Susceptibility loci for adiposity phenotypes on 8p, 9p, and 16q in American Samoa and Samoa. Obesity (Silver Spring, Md).

[21] Vogel MJ, van Zon P, Brueton L, Gijzen M, van Tuil MC, Cox P (2012). Mutations in GRIP1 cause Fraser syndrome. J Med Genet.

[22] Geiger JC, Lipka J, Segura I, Hoyer S, Schlager MA, Wulf PS (2014). The GRIP1/14-3-3 pathway coordinates cargo trafficking and dendrite development. Dev Cell.

[23] Mejias R, Adamczyk A, Anggono V, Niranjan T, Thomas GM, Sharma K (2011). Gain-of-function glutamate receptor interacting protein 1 variants alter GluA2 recycling and surface distribution in patients with autism. Proc Natl Acad Sci U S A.

[24] Balaci L, Spada MC, Olla N, Sole G, Loddo L, Anedda F (2007). IRAK-M is involved in the pathogenesis of early-onset persistent asthma. Am J Hum Genet.

[25] Hulsmans M, Geeraert B, De Keyzer D, Mertens A, Lannoo M, Vanaudenaerde B (2012). Interleukin-1 receptor-associated kinase-3 is a key inhibitor of inflammation in obesity and metabolic syndrome. PLoS One.

[26] Ahmed ZM, Yousaf R, Lee BC, Khan SN, Lee S, Lee K (2011). Functional null mutations of MSRB3 encoding methionine sulfoxide reductase are associated with human deafness DFNB74. Am J Hum Genet.

[27] Lee E, Kwak GH, Kamble K, Kim HY (2014). Methionine sulfoxide reductase B3 deficiency inhibits cell growth through the activation of p53-p21 and p27 pathways. Arch Biochem Biophys.

[28] Chen J, Meng Y, Zhou J, Zhuo M, Ling F, Zhang Y (2013). Identifying candidate genes for type 2 diabetes mellitus and obesity through gene expression profiling in multiple tissues or cells. J Diabetes Res.

[29] Yu NK, Kim HF, Shim J, Kim S, Kim DW, Kwak C (2016). A transducible nuclear/nucleolar protein, mLLP, regulates neuronal morphogenesis and synaptic transmission. Sci Rep.

[30] Guler GD, Liu H, Vaithiyalingam S, Arnett DR, Kremmer E, Chazin WJ (2012). Human DNA helicase B (HDHB) binds to replication protein a and facilitates cellular recovery from replication stress. J Biol Chem.

